# Presentation and management of neural tube defects in the middle belt of Ghana

**DOI:** 10.1007/s00381-025-06869-3

**Published:** 2025-06-05

**Authors:** Joseline Haizel-Cobbina, Samuel Addy, Derrick Obiri-Yeboah, Kwadwo Darko, Darell A. Addison, Megan E. H. Still, Kwaku Ampofo, Christian Coompson, Dickson Bandoh, Benedict Owusu, Anthony Lamina, Jeffrey P. Blount, James M. Johnston, Michael C. Dewan, Frank Nketiah-Boakye

**Affiliations:** 1 Department of Neurological Surgery, Vanderbilt University Medical Center, Nashville, TN USA; 2 Vanderbilt Institute for Global Health, Vanderbilt University Medical Center, 2525 West End Avenue, Nashville, TN 37203 USA; 3 Department of Surgery, Neurosurgery Unit Komfo Anokye Teaching Hospital, Kumasi, Ghana; 4 Department of Neurosurgery, Mayo Clinic College of Medicine and Science, Rochester, MN USA; 5 Department of Neurosurgery, University of Florida, Gainesville, FL USA; 6 Division of Pediatric Neurosurgery, Children’s of Alabama, University of Alabama at Birmingham, Birmingham, AL USA

**Keywords:** Neural tube defect, Spina bifida, Low- and middle-income countries, Folate fortification, Pediatric neurosurgery, Capacity strengthening

## Abstract

**Introduction:**

Neural tube defects (NTDs) have a high incidence of morbidity and mortality in resource-limited countries. Here, we determine the birth prevalence and describe the presentation, diagnosis, and management of NTDs at Komfo Anokye Teaching Hospital (KATH) in Ghana.

**Methods:**

An ambispective study was conducted for all patients presenting to KATH with NTD from 2019 to 2023. Descriptive statistics and geospatial analysis were completed for relevant clinical data including patient demographics, clinical presentation, surgical details, treatment outcomes, and travel distance.

**Results:**

One hundred seventy-one patients presented with NTDs at a median age of 24 days [IQR 6, 113]. Birth prevalence was 4.7 per 1000 births. The majority of patients presented with myelomeningocele (MM) (79%) followed by encephalocele (16%). Surgical repair was performed for 131 (97%) MM cases at a median age of 33 days [IQR: 18–94 days] and for 25 (93%) encephalocele cases at a median age of 183 days [IQR: 41–384 days]. The median post-operative length of stay was 12 days [IQR 6, 22]. The surgical mortality rate was 7% (11/163). 76 (55%) MM patients developed hydrocephalus, of which 24 (32%) underwent CSF diversion at a median interval of 36 days [IQR 21, 143] following MM repair. Follow-up data were available for 158 (92%) patients, with a median follow-up duration of 16 months (IQR: 4–29 months). At the last follow-up, 70% of patients were alive.

**Conclusion:**

Timely and affordable neurosurgical care remains a challenge for NTD patients at KATH and may contribute to elevated morbidity and mortality.

## Introduction

Neural tube defects (NTDs) are congenital anomalies of the central nervous system characterized by malclosure of the rostral and/or caudal neuropore during early embryogenesis giving rise to cranial or spinal dysraphism [1, 2]. They may present as open defects such myelomeningocele and craniorachischisis, or closed defects such as encephalocele and spina bifida occulta [2, 3]. The etiology of NTDs is complex and multifactorial, involving both genetic and environmental factors. Mutations in genes related to folic acid uptake and metabolism, maternal folate deficiency, and maternal exposure to teratogens all can increase the risk of NTDs [4, 5].

Despite the global decline in the prevalence of NTDs, the birth prevalence of NTDs is far greater in low- and middle-income countries (LMICs) (37.6/10,000 births) than in high-income countries (7.6/10,000 births) [6, 7]. Moreover, there are significant data gaps from LMICs including Ghana due to the absence of national surveillance systems or comprehensive epidemiological studies to provide true prevalence estimates.

While most NTDs are nonfatal, they are associated with impaired neurological function, with open defects generally leading to worse functional neurosurgical outcomes compared to closed defects [2]. Hydrocephalus and Chiari II malformation are important comorbidities that impact NTDs outcomes. For myelomeningoceles, the Congress of Neurological Surgeons currently recommends a repair of the defect within the first 48 h of birth to prevent further neurological deterioration [8, 9]. However, in resource-limited countries, morbidity and mortality remain high largely due to challenges of timely presentation, poor neurosurgical infrastructure, and lack of long-term multidisciplinary team care [10].

The prevalence, neurosurgical management and outcomes of NTDs in Ghana are poorly understood. Herein we describe the birth prevalence, presentation, and outcomes following management of NTDs at a major teaching hospital in the middle belt of Ghana.

## Methods

### Design and setting

This ambispective cohort study was conducted at Komfo Anokye Teaching Hospital (KATH) in Kumasi, Ghana. KATH, one of eleven hospitals in Ghana providing neurosurgical services to adults and children [11]. The study received ethical clearance from the institutional review boards (IRBs) of KATH and Vanderbilt University Medical Center (VUMC).

KATH is the second largest major public tertiary and referral facility in Ghana covering 10 out of the 16 regions in the country with a catchment population of 14.9 million people [12]. Five neurosurgeons at KATH offer care to adults and children, including one with an interest in establishing a pediatric-focused practice.

### Data collection

The study reviewed all delivery records and newborn referral records at the Obstetrics and Gynecology Department and Mother and Baby Unit respectively between 2019 and 2023. The KATH Neurosurgery Unit has maintained a hydrocephalus and spina bifida (HSB) Research Electronic Data Capture (REDCap) database since 2019 after the implementation of the CURE Neuro Program in Kumasi Ghana. This program was implemented after one neurosurgeon at KATH (FNB) completed the CURE International Hydrocephalus and Spina Bifida (CHSB) fellowship training program in Uganda during which time he underwent training for the endoscopic management of hydrocephalus [13]. The REDCap database incorporates patient data on clinical presentation, treatment offered, follow-up, and outcomes for neural tube defects and hydrocephalus. The HSB clinical coordinator reviewed patient medical charts after clinic visits and in-patient hospital stays and entered data in the database. Patients who missed clinic appointments were followed up via home visits and/or telephone calls.

For this study, the HSB database was reviewed with data collected on all pediatric patients (0–19 years) who were diagnosed managed at KATH for NTDs between 2019 and 2022. Data on new patients diagnosed and managed in 2023 as well as follow-up data on existing patients were prospectively collected. We reviewed data on patient demographics, type of NTD, condition of NTD on initial presentation, age at diagnosis, management, co-morbid conditions, age at the time of repair, post operative complications, length of hospital stay, surgical mortality, and follow-up. Surgical mortality was defined as death within 30 days of surgical intervention.

### Statistical analysis

Descriptive statistics, including median and interquartile ranges for continuous variables and counts and frequencies for categorical variables, were utilized to summarize the dataset. To estimate the hospital incidence of NTDs, we reviewed all newborn delivery records at KATH and newborn referrals from the KATH catchment area during the study period. Birth prevalence was calculated by dividing the aggregate number of NTDs by the total number of newborn deliveries including live births and stillbirths and external newborn referrals and expressed per 1000 births.

A geospatial analysis was conducted to assess the distances between KATH and the locations of patient residence using spatial data visualization. The hospital’s coordinates were manually defined, while location data, including longitude and latitude, were defined for each patient’s location. The Haversine formula, implemented through the geosphere package, was used to compute geodesic distances in kilometers (km) between the hospital and each location [14]. The results of this analysis were represented on a Ghana map using the ggplot package in R studio.

Univariate linear regression analyses were performed to explore whether there was an association between travel distance and (1) age at presentation and (2) time to surgical intervention. The dataset was imputed using the k-nearest neighbors imputation method (*k* = 5) to handle missing data for regression analysis. We reported beta coefficient along with 95% confidence intervals (CI) and *p*-value. A two-tailed *p* < 0.05 was the set threshold for statistical significance. Data analysis was performed using Microsoft Excel (Version 2209) and R Studio (Version 4.3).

## Results

### Demographics and clinical presentation

**Fig. 1:**
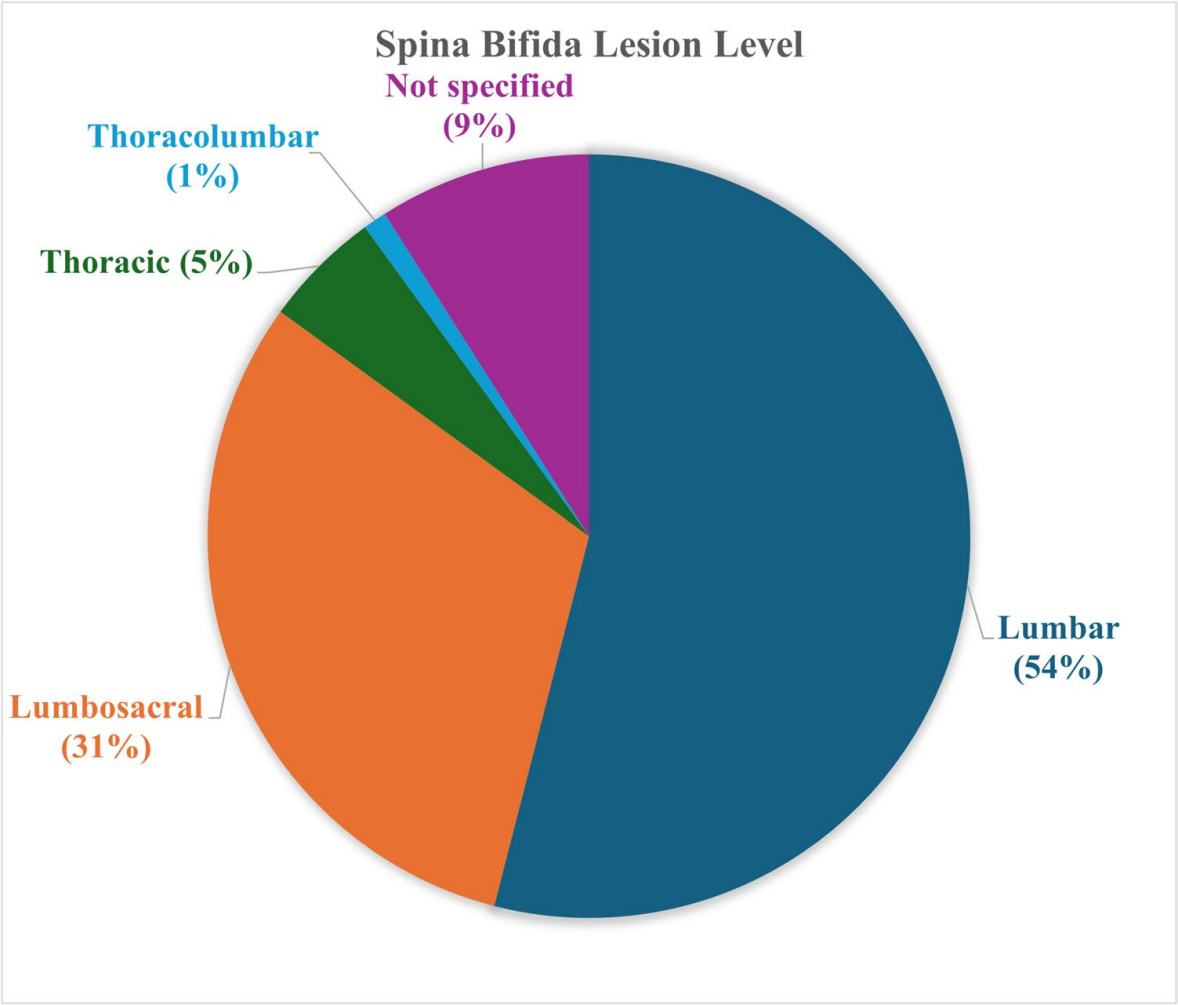
A pie chart representation of the distribution of spina bifida lesion level among pediatric patients

A total of 28,994 deliveries and 7,487 external referrals were recorded at KATH with 171 patients diagnosed with NTDs. The estimated birth prevalence over the 5-year period was 4.7 per 1000 births. The median age at the time of diagnosis was 24 days [IQR 6, 113]. Fifty-one percent were females. The majority of patients presented with myelomeningocele (MM) (*n* = 135, 79%) followed by encephalocele (*n* = 27, 16%) (Table 1). The remaining patients presented with tethered cord (*n* = 4, 2%), meningocele (*n* = 3, 2%), and lipomeningocele (*n* = 2, 1%). Majority of spinal dysraphism patients had a lumbar lesion (*n* = 76, 54%) (Fig. 1). Among the MM patients, 16 (12%) presented with a ruptured sac and another 6 (4%) with an infection at the site of the lesion. Occipital encephalocele (*n* = 26, 96%) was the most common type of encephaloceles encountered; 4% were nasofrontal encephaloceles. Two of the occipital encephaloceles were labelled as infratorcular and 24 were labelled as supratorcular. Table 1 summarizes patient demographics and clinical presentation.

**Table 1:** Demographics and clinical characteristics of patients with NTDs

Variable	Number (%)
**Age at presentation for all NTDs (in days)**	
Median (IQR)	24 (6, 113)
**Age at presentation for myelomeningocele (in days)**	
Median (IQR)	21 (6, 73)
**Gender (all NTDs)**	***N* = 171**
Female	84 (49.1%)
Male	87 (50.9%)
**Gender breakdown for MMCs**	***N* = 135**
Female	63 (45.7%)
Male	72 (53.3%)
**Type of NTDs**	***N* = 171**
Myelomeningocele	135 (78.9%)
Encephalocele	27 (15.8%)
Tethered cord	4 (2.3%)
Meningocele	3 (1.8%)
Lipomyelomeningocele	2 (1.2%)
**Condition of myelomeningocele on presentation**	***N* = 135**
Ruptured	16 (11.9%)
Infected	6 (4.4%)

*NTDs*, neural tube defects; *MMC*, myelomeningocele

### Surgical repair of NTDs

Overall, 163 (94%) NTDs underwent surgical repair at a median age of 33 days [IQR, 18, 94]. Of these, 131 (97%) patients with MMs underwent surgical repair at a median age of 31 days [IQR, 18, 73] and 23 (85%) encephaloceles were repaired at a median age of 183 days [IQR, 41, 384]. The median time from presentation to surgical management was 15 days [IQR, 6, 36].

Post NTDs repair, wound breakdown requiring wound revision (*n* = 12, 7%) and surgical site infection (*n* = 7, 4%) were the most common surgical complications. The median post-operative length of hospital stay was 12 days [IQR, 6, 22] (Table 2). Surgical mortality was 7%.

**Table 2:** Surgical details and follow-up

**Myelomeningocele**	***N***= **135**
Repair alone	107 (79%)
Repair + ETV	4 (3%)
Repair + ETV/CPC	3 (2%)
Repair + VPS	17 (13%)
No repair	4 (3%)
**Encephalocele**	***N***= **27**
Repair alone	20 (74%)
Repair + ETV/CPC	3 (11%)
Repair + VPS	2 (7%)
No repair	2 (7%)
Other defects*	***N***= **9**
Repair	9 (100%)
**Post-operative LOS, median [IQR] (days)**	**12 **[6, 22]
**Post-operative complications**	***N***= **163**
Wound breakdown/revision	12 (7%)
Surgical site infection	7 (4%)
CSF leak	2 (1%)
Other	3 (2%)
Surgical mortality	11 (7%)
Hospital readmission	6 (4%)
Follow-up	*N* = 158
*Clinical state*	
Alive	110 (70%)
Mortality	48 (30%)
**Length of follow-up, median [IQR] (months)**	16 [4, 29]

*Tethered cords, meningoceles, and lipomyelomeningoceles

*CSF*, cerebrospinal fluid; *ETV*, endoscopic third ventriculostomy; *CPC*, choroid plexus cauterization; *IQR*, interquartile range *LOS*, length of hospital stay; *VPS*, ventriculoperitoneal shunt

### Hydrocephalus treatment

Among the 135 MM patients, 76 (55%) developed hydrocephalus out of which 24 (32%) patients underwent CSF diversion. Out of the 24 patients who were treated for hydrocephalus, 2 (8%) patients underwent CSF diversion at the time of MM repair and 22 patients (92%) underwent CSF diversion at a median time of 69 days [IQR, 24, 144] after MM repair. A majority (*n* = 17) of these patients underwent a ventriculoperitoneal shunt (VPS) insertion and 7 patients underwent ETV ± CPC.

Among the 27 encephalocele patients, 5 (19%) received treatment for hydrocephalus. Four patients underwent CSF diversion at a median time of 56 days [IQR 38, 100] post-surgical repair and 1 patient underwent CSF diversion 2 days before the surgical repair was done.

### Follow-up

Median follow-up duration for 158 (92%) patients with follow-up data was 16 months (IQR 4, 29). 70% of patients with follow-up data were alive at last follow-up.

### Geographic distribution and travel distances

**Fig. 2:**
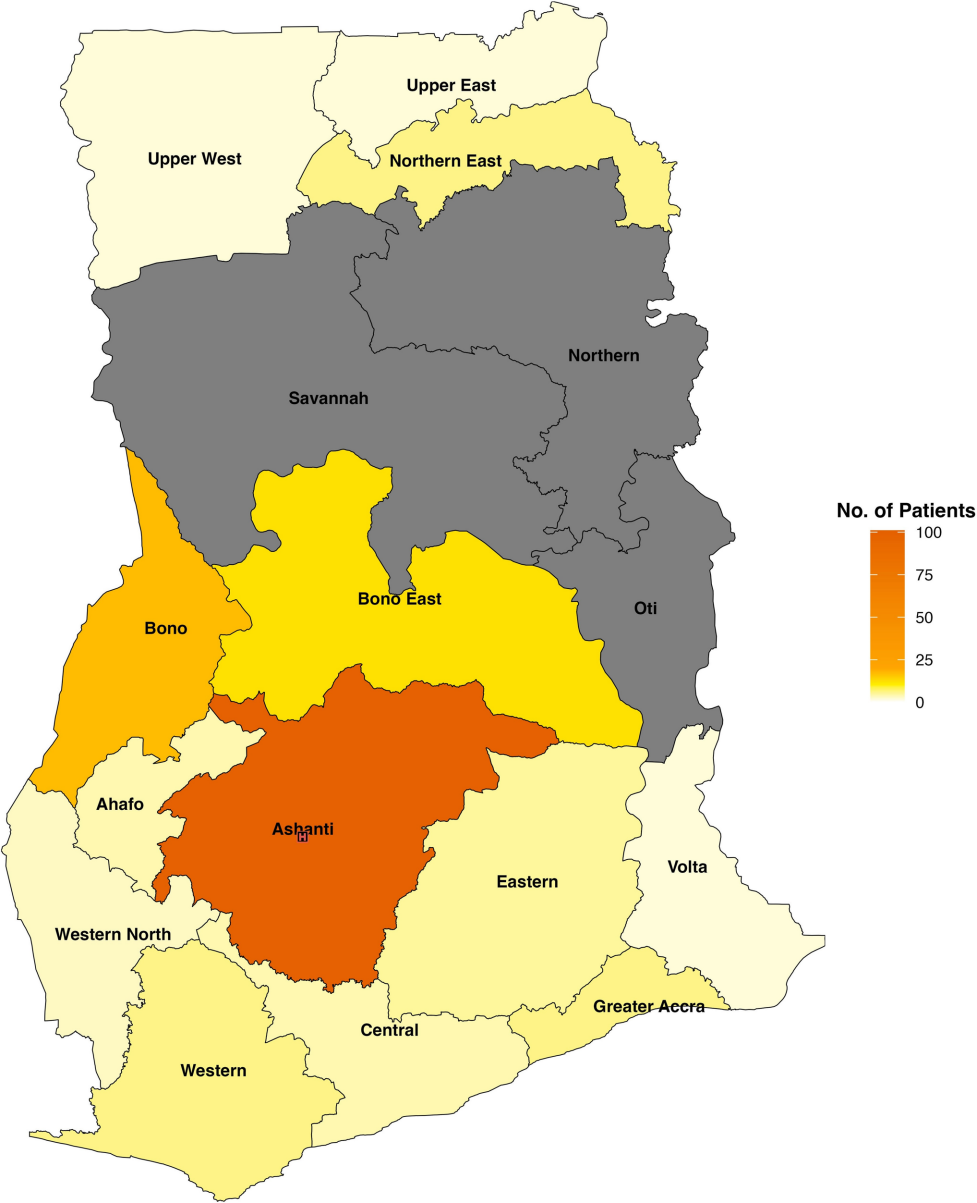
A map of Ghana showing the geographic distribution of pediatric patients with neural tube defects seeking pediatric neurosurgical care at Komfo Anokye Teaching Hospital in Kumasi, Ashanti Region

**Fig. 3:**
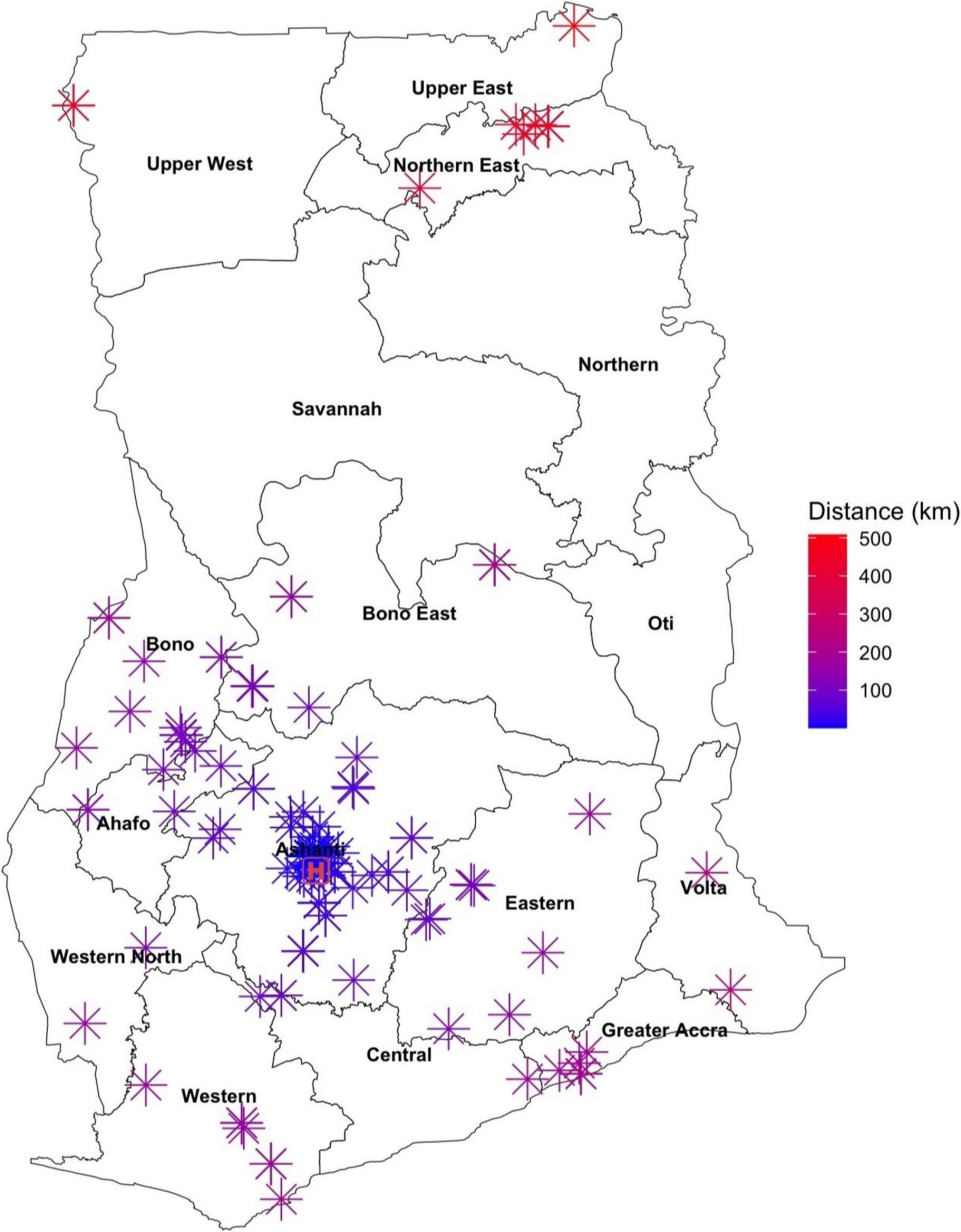
A map showing the geospatial distribution of travel distances of pediatric patients with neural tube defects seeking pediatric neurosurgical care at Komfo Anokye Teaching Hospital in Kumasi, Ashanti Region

Geographic analysis revealed that most patients (*n* = 101, 59%) seeking pediatric neurosurgical care at KATH resided within the Ashanti Region where KATH is located. The remaining 41% (*n* = 70) of patients traveled from 12 other regions in the country to access care at KATH (Fig. 2). Patients residing in the Ashanti Region traveled a median distance of 12 km [IQR, 0.3, 38] to KATH (Fig. 3). For patients residing outside of Ashanti Region, the median distance traveled was 133 km [IQR, 104, 198] with the longest recorded journey of 508 km from Bawku to Kumasi. For the entire cohort, the median travel distance to access care at KATH was 55 km [IQR, 6, 129]. Univariate linear regression demonstrated that the length of travel was not significantly associated with age at presentation (*β* = 0.01, 95% CI − 0.01 to 0.03, *p* = 0.45) and time to surgical intervention (*β* = 0.02, 95% CI − 0.09 to 0.12, *p* = 0.76).

## Discussion

This study examines the management and outcomes of 171 patients with NTDs in Kumasi, Ghana over a 5-year period. The estimated birth prevalence of 4.7 per 1000 births in the current study is relatively higher than what has been reported in previous Ghanaian hospital-based studies (1.15–1.6 per 1000 births) but similar to the overall prevalence in Africa and India (4.5–5.1 per 1000 births) [15–18]. Myelomeningocele was the most commonly managed NTD. A majority of patients underwent a surgical repair with a 7% surgical mortality reported similar to rates reported in Zambia, Ethiopia, and Turkey (7–8%) [10, 19, 20]. Follow-up revealed a 30% mortality in our cohort similar to a Ugandan cohort (34%) [21].

We noted significant delays in both presentation and receiving neurosurgical intervention. Currently in Ghana, pediatric neurosurgical care is largely concentrated in 5 major tertiary facilities in three regions with patients traveling long distances for care [11, 12]. While the geospatial analysis provides an approximation of the travel distances between patient location and KATH, these geodesic figures understate the true travel burden for most patients. There may be other significant factors beyond travel distance which may have contributed to delays in seeking care. These include but not limited to the cost of transportation and lodging, health literacy, and a poor referral system which need to be explored in future studies [22, 23]. Our results also showed that patients with MMC presented earlier than encephalocele patients which could be related to parents/caregivers perceived severity of MMC possibly due to observed complications in the newborn including limb weakness and deformities [24]. As a consequence of the delayed access to specialized care, about 16% of MMC patients presented with an infected or ruptured lesion.

After patients present at the hospital, several systemic and logistical challenges further contribute to delays in receiving neurosurgical intervention. Pediatric patients compete for care with adults as there are currently no dedicated pediatric clinics, operating rooms, or pediatric neurosurgeons [12]. This, coupled with the limited neurosurgical infrastructure, impacts the timely delivery of care. As such, the timing of surgical repair was largely dependent on availability of OR space and equipment. Additionally, medical stabilization for those who presented with rupture or infection may have been required prior to surgery, which may have further prolonged the wait time to neurosurgical repair and postoperative hospital stay [25].

In this study cohort, 55% of MM patients and 19% of encephalocele patients developed hydrocephalus, comparable to previous African reports (50–54%) [10, 19, 26]. Among patients who developed hydrocephalus, only about a third underwent CSF diversion despite the availability of both ventriculoperitoneal shunt (VPS) and endoscopic third ventriculostomy with or without choroid plexus cauterization (ETV/CPC) at KATH. Anecdotally, some of the major reasons cited by caregivers for not seeking follow-up hydrocephalus treatment or declining hydrocephalus treatment despite counselling included socioeconomic constraints and sociocultural and spiritual beliefs. Some patients also died while waiting for their families to raise funds for CSF diversion due to the cost of care. A tragic case involved a mother who reportedly committed suicide due to challenges with taking care of a child with a disability and the associated social stigma. This resulted in the inability of the child to follow through with care as identifying a relative willing to assume responsibility for the child’s care proved challenging. Unfortunately, such occurrences are common in African communities where mothers are often blamed for a child having a disabling condition, and in some instances, the child is also subjected to abuse and neglect [27, 28].

Research has shown that maternal periconceptional folic acid supplementation can prevent 70% of NTDs. However, a previous Ghanaian study showed that about 75% of pregnant women surveyed reported never taking folate supplements or consuming folate fortified food products during the periconceptional period [29]. Food fortification initiatives in Ghana date back to 1996; however, efforts to fortify food products with folate have faced significant challenges and are currently limited to wheat flour only [30].

To reduce the incidence of NTDs and improve care access for affected patients, a systems approach is needed. This requires both awareness campaigns to promote folate consumption and a legislative action to implement a mandatory folate fortification policy and a public health education to promote folate consumption, correcting misconceptions and improving health-seeking behaviors [29]. Improving access to care and patient outcomes and achieving a timely NTD repair will require strengthening pediatric neurosurgical care capacity in Ghana. This could be achieved through creating neurosurgical training and pediatric neurosurgery fellowship opportunities to increase the workforce and equipping tertiary hospitals with the needed neurosurgical infrastructure, including dedicated pediatric operating room spaces. Establishing NTDs multidisciplinary clinics and efficient communication with peripheral health facilities will help ensure timely referrals after childbirth, and more consistent pregnancy care could help identify these patients prior to birth, further improving referral times. To help support patient care, KATH in partnership with the Spina Bifida and Hydrocephalus Foundation of Ghana and Child Help International has currently established a free hostel facility named “House of Hope” mainly as a centre for therapy and for caregivers traveling long distances to KATH for care. The cost of hardware is catered for by Child Help International to help alleviate the cost of care. One of the neurosurgeons (FNB) has undergone a pediatric neurosurgery clinical observership program at University of Alabama at Birmingham (UAB), USA, and has a strong interest in establishing a pediatric neurosurgery practice at KATH. Together, these initiatives not only seek to reduce the burden of NTDs in Ghana but also ensure holistic support for affected children and their families.

## Limitations

There are a number of study limitations to consider. Despite efforts to bridge gaps in follow-up with telephone calls and home visit, close to 10% of patients were still missing follow-up data. Also, information of the cause of death for patients who died during the follow-up was not available. The non-existence of multidisciplinary team care also made it challenging to obtain data on non-neurosurgical complications and its impact on morbidity and mortality. Despite these limitations, we believe the findings of the study provide valuable insight into the surgical outcomes in our setting and the pitfalls with follow-up care and surveillance. While the study findings and recommendations made will serve as a catalyst for a systems-level change aimed at decreasing NTDs burden and improving outcomes in Ghana, it is imperative that future research endeavors include a more detailed assessment of the predictors of mortality and morbidity.

## Conclusion

This study is the first to characterize the NTD population at KATH and provide insight on management outcomes. Timely and affordable surgical and follow-up care remains a challenge and may contribute to elevated morbidity and mortality relative to better-resourced healthcare systems. The study findings may lead to practical efforts aimed at both preventing NTDs and improving treatment outcomes.

## Data Availability

No datasets were generated or analysed during the current study.
